# Cultural adaptation of health interventions including a nutrition component in Indigenous peoples: a systematic scoping review

**DOI:** 10.1186/s12939-021-01462-x

**Published:** 2021-05-22

**Authors:** Lisa Vincze, Katelyn Barnes, Mari Somerville, Robyn Littlewood, Heidi Atkins, Ayala Rogany, Lauren T. Williams

**Affiliations:** 1 School of Health Sciences and Social Work, Griffith University, Parklands Drive, Gold Coast, Queensland 4222 Australia; 2 Menzies Health Institute Queensland, Griffith Health Centre, G40_8.86, Gold Coast, Queensland 4222 Australia; 3 Academic Unit of General Practice, Medical School, College of Health & Medicine, The Australian National University, Canberra, Australian Capital Territory 2601 Australia; 4 Health & Wellbeing Queensland, Queensland Government, Brisbane, Queensland Australia; 5 Queensland Child and Youth Clinical Network, Clinical Excellence Queensland, Queensland Government, Brisbane, Queensland Australia; 6 Queensland Children’s Hospital, Children’s Health Queensland Hospital and Health Service, Queensland Government, Brisbane, Queensland Australia

**Keywords:** Aboriginal, Adult, Child, Co-design, Cultural safety, Community, Indigenous, First nations

## Abstract

**Background:**

Indigenous populations throughout the world experience poorer health outcomes than non-indigenous people. The reasons for the health disparities are complex and due in part to historical treatment of Indigenous groups through colonisation. Evidence-based interventions aimed at improving health in this population need to be culturally safe. However, the extent to which cultural adaptation strategies are incorporated into the design and implementation of nutrition interventions designed for Indigenous peoples is unknown. The aim of this scoping review was to explore the cultural adaptation strategies used in the delivery of nutrition interventions for Indigenous populations worldwide.

**Methods:**

Five health and medical databases were searched to January 2020. Interventions that included a nutrition component aimed at improving health outcomes among Indigenous populations that described strategies to enhance cultural relevance were included. The level of each cultural adaptation was categorised as evidential, visual, linguistic, constituent involving and/or socio-cultural with further classification related to cultural sensitivity (surface or deep).

**Results:**

Of the 1745 unique records screened, 98 articles describing 66 unique interventions met the inclusion criteria, and were included in the synthesis. The majority of articles reported on interventions conducted in the USA, Canada and Australia, were conducted in the previous 10 years (*n* = 36) and focused on type 2 diabetes prevention (*n* = 19) or management (*n* = 7). Of the 66 interventions, the majority included more than one strategy to culturally tailor the intervention, combining surface and deep level adaptation approaches (*n* = 51), however, less than half involved Indigenous constituents at a deep level (*n* = 31). Visual adaptation strategies were the most commonly reported (*n* = 57).

**Conclusion:**

This paper is the first to characterise cultural adaptation strategies used in health interventions with a nutrition component for Indigenous peoples. While the majority used multiple cultural adaptation strategies, few focused on involving Indigenous constituents at a deep level. Future research should evaluate the effectiveness of cultural adaptation strategies for specific health outcomes. This could be used to inform co-design planning and implementation, ensuring more culturally appropriate methods are employed.

**Supplementary Information:**

The online version contains supplementary material available at 10.1186/s12939-021-01462-x.

## Background

Indigenous peoples are recognised as being connected to a particular geographical region and having ancestral ties to the original land inhabitants prior to the development of modern states and borders [1, 2]. Indigenous peoples share unique cultural, societal, environmental, political and economic values that differ from the dominant society in which they live [1, 3]. Despite having strong ancestral connections to original land owners, Indigenous peoples have consistently faced marginalization and the denial of basic human rights and represent about one third of the world’s poorest communities [4]. The consequences of marginalisation and poverty include significantly poorer health outcomes [5] and reduced access to quality education and health services [2, 4, 6]. Persistent institutional racism further contributes to the health gap between Indigenous and non-indigneous people [7]. Clearly, action is required to address the health disparities faced by Indigenous peoples.

Improving diet quality has been shown to reduce the incidence of chronic diseases by up to 50% [8]. Therefore, effective nutrition interventions for combatting the gaps in health outcomes, particularly preventable chronic diseases, are needed for Indigenous peoples. While there is some evidence that nutrition interventions can improve diet-related health outcomes in Indigenous populations [9], a systematic review of 26 nutrition-related interventions among Australian Aboriginal and Torres Strait Islander people showed these gains to be short-term [10]. The authors concluded that lack of cultural adaptation limited the long-term effectiveness of interventions.

Indigenous peoples hold an holistic view of health incorporating community, environment, spiritual, emotional and physical wellbeing [11, 12]. This is in contrast to the typical western model of health care provision which is more individualistic and disease-centric [13]. A recent systematic scoping review of Indigenous primary healthcare service delivery models identified a preference for healthcare that was accessible, culturally appropriate, holistic and involved community participation [14]. While all interventions need to be tailored, this is particularly true for Indigenous peoples, acknowledging their unique cultural needs and healthcare preferences.

Ensuring cultural safety of evidence-based interventions to improve health is therefore an important approach [15, 16]. Curtis and colleagues propose a comprehensive definition for cultural safety that references the need for *“healthcare organisations to influence healthcare to reduce bias and achieve equity”* [17]. Cultural adaptation of an intervention involves careful consideration of the needs of the group for whom the intervention is being developed, as well as a meaningful collaboration during intervention design, development, implementation and evaluation [15, 18]. Frameworks to achieve cultural adaptation have been developed. Kreuter and colleagues [19] describe five categories of adaptation commonly used to make health interventions more culturally appropriate (evidential, visual, linguistic, constituent involving, and socio-cultural). Resincow et al. [20] further posits that cultural sensitivity in developing interventions consists of two dimensions: surface (gives the sense of culturally appropriate messages reflecting settings and experiences of the group, including: music, pictures, foods, clothing, locations, and people) or deep (involves cultural sensitivity and a comprehensive understanding of the ethnic group’s core cultural values, norms, and stressors affecting health behaviours).

The type and level of cultural adaptation strategies incorporated into the design and implementation of nutrition interventions for Indigenous peoples is unknown. According to Munn and colleagues [21], a scoping review is the type of review indicated when the aim is to identify key characteristics from an evidence base. This review therefore aims to examine the range of research undertaken on nutrition-related health interventions that are culturally adapted for Indigenous peoples focusing on the type and nature of adaptations made.

## Methods

A scoping review was conducted systematically using a predefined protocol following the methodological framework of Arksey and O’Malley [22]. This approach included identifying the research question, selecting studies relevant to the research question, and charting the data - which includes summarising and reporting the results. This review was conducted in accordance with the Preferred Reporting Items for Systematic Reviews- Scoping reviews extension checklist [23].

### Identifying the research question

This review aimed to identify and categorise the key characteristics of cultural tailoring in health interventions with a nutrition component designed for Indigenous populations. The system outlined by the United Nations was adopted to determine Indigenous populations from any nation worldwide [24], noting that terms vary by country and geographical region and that the right to identify as Indigenous is the right of the people themselves [25]. Interventions conducted in mixed populations (i.e. Indigenous and non-indigenous peoples) were excluded.

Lifestyle interventions with a nutrition component with the aim of improving health outcomes, that described deliberate strategies used to enhance cultural relevance were included. Nutrition components of interventions were defined as the manipulation of food or dietary intake directly (e.g. provision of food or nutritional supplement) or indirectly (e.g. nutrition education). The nutrition component could be the sole focus of the intervention or delivered in conjunction with other components such as physical activity.

The typology of Kreuter and colleagues [19] was used to categorise the cultural adaptation strategies (Table 1). Articles were included if they described at least one of the five strategies. To further explore the extent of cultural adaptations, the model for understanding cultural sensitivities of Resincow and colleagues was also applied to included studies [20]. Each strategy was classed as ‘surface’ or ‘deep’ according to this model [20] (Table 1).

**Table 1:** Description of cultural adaptation strategies used to tailor nutrition health interventions for Indigenous peoples

Strategy type	Strategy Description^a^	Sensitivity Level^b^
Peripheral	**Use of colours, imagery, fonts, pictures of the community, music, or declarative titles.** Gives the *appearance* of cultural appropriateness by packaging them in ways likely to appeal to a group. These elements can create interest, establish credibility and set the tone for content in printed communications	Surface
Evidential	**Use of data on a given health issue within the population/community.** To enhance the perceived relevance of a health issue for a given group by presenting evidence of its impact on that group. Such statements seek to raise awareness, concern, and or perceived personal vulnerability to a health issue by showing that it affects the given group.	Surface
Linguistic	**Use of dominant or traditional language.** To make programs and materials more accessible. Must consider translations – should be culturally relevant, not direct translations.	Surface (direct translation) Deep (full translations with culturally relevant statements)
Constituent Involving	**Drawing directly on the experience of members of the community.** Can range from using stories of community members, through to formal community guidance, through to full ownership and directing of the project by the community.	Surface (testimonials or stories) Deep (delivery of intervention by community members)
Sociocultural strategies	**Discusses health issues in the context of broader social and cultural values and characteristics.** The cultural values, beliefs and behaviours of the group are recognised, reinforced and built upon to provide context and meaning to the health promotion activity.	Deep

Adapted from: ^a^Kreuter et al., 2003 [19] and ^b^Resincow et al., 1999 [20]

Published peer-reviewed studies, of any design, were considered for inclusion. Articles were included if the health outcomes were reported or if the health outcomes were specified but not yet evaluated within the existing publication. Where health outcomes were not stated, a category of ‘no outcomes reported’ was used. The health outcomes of interest were reported in the description of each included study (Additional file 1). Where health outcome data was available, the specific changes were extracted (e.g. decreased weight, increased intake of vegetable serves).

### Selection of studies relevant to the research question

A structured search of available peer-reviewed literature was conducted with support from an experienced health librarian. The following databases were searched from inception to January, 2020: Embase, Cumulative Index to Nursing and Allied Health Literature (CINAHL), Cochrane Central Register Medline, PsychInfo, and Scopus (restricted to non-Medline indexed articles). Terms searched can be seen in Additional file 2. A web application (Rayaan) was used to manage the review (Available at: https://rayyan.ai/) [26]. All included studies were hand searched for references not captured by the initial search strategy. Reference lists of identified systematic reviews were searched to check all relevant papers from those reviews were included. Title/abstract and full-text screening were coded independently in duplicate. Discrepancies were resolved through discussion.

### Charting of data

Data from all included articles were extracted into a purposefully-developed spreadsheet by one researcher. A second researcher reviewed the coding on a subsample of articles (*n* = 24; ~ 25%) to ensure adequate and consistent application of the inclusion and exclusion criteria. Extracted data included: first author name and year of publication, intervention or program name, Indigenous population name (cited as reported in the article including any tribal affiliations) and location (including country), population of interest, level of intervention (individual (aimed at individual behaviour change), community (aimed at community behavioural or value change), or systems (aimed at environmental change (e.g. food systems)), a brief description of the intervention (including length, frequency and duration), theories of behaviour change used to underpin the intervention, a brief description of intervention outcomes (if reported), and whether or not formative research was undertaken in the same population or community (i.e. evidence for the intervention to improve health outcomes for the people involved). To achieve a yes for formative research, authors needed to refer to evidence (published or unpublished) to support the application of the intervention or program within the Indigenous population or specific community they were planning to work with. Involvement of the community in intervention design was extracted as ‘yes-minimal’ if community members were reported to have been consulted or asked to provide feedback, ‘yes-maximal’ if community-based participatory research was employed, and ‘no’ if there was no community involvement in design of the intervention or program. Categories of strategies used to culturally tailor the intervention were extracted as per Table 1 [19].

Where multiple articles clearly reported on the same intervention and focussed on health outcomes, they were grouped for data extraction. As per the convention of scoping reviews, extracted data was summarised numerically to provide an overview of study characteristic frequency.

## Results

**Fig. 1:**
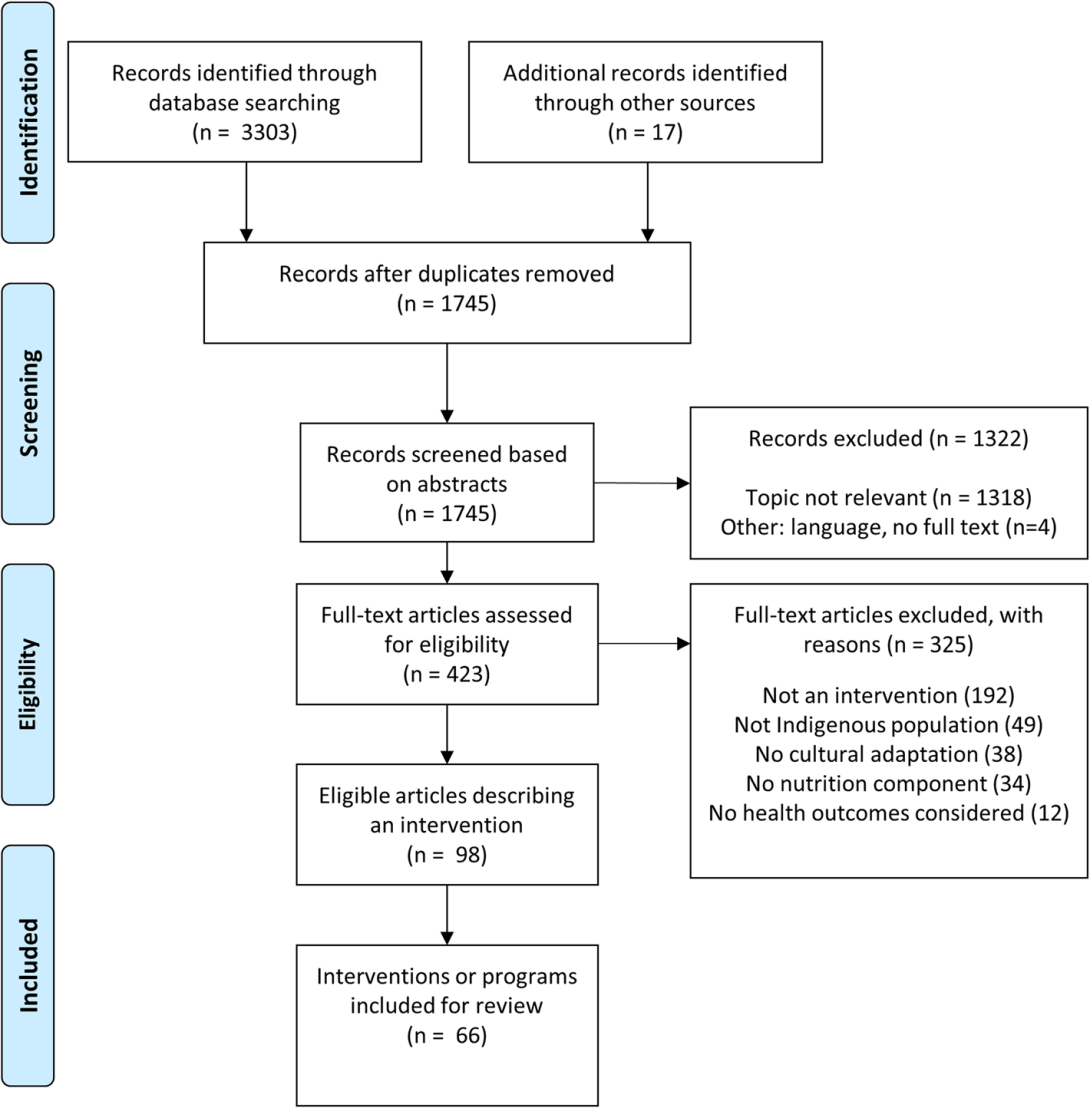
Flow diagram for identification of culturally adapted health interventions for Indigenous peoples

The database search resulted in 3303 records with an additional 17 articles identified through other sources (Fig. 1). There were 1745 Articles remaining once duplicates were removed. Of the 423 articles included in full text screening, 98 met the inclusion criteria. These 98 records described 66 unique interventions. The articles described 19 randomised controlled trials (RCTs), 3 Randomised Trials, 9 non-randomised trials and 35 pre-post studies.

### Characteristics of included studies

Table 2 summarises the characteristics of included interventions (*n* = 66) [27–92]. Individual study characteristics are described in Additional file 2. Where an intervention has multiple articles, the first publication by date has been used throughout as the source reference.

**Table 2:** Characteristics of the 66 included interventions

Study Characteristic	n	(%)
*Population (Country)*		
Native American / Native Alaskan / Native Hawaiian (USA)	33	50.0
Aboriginal and/or Torres Strait Islander People (Australia)	14	21.2
First Nations Peoples (Canada)	11	16.8
Maori (New Zealand)	4	6.0
Other Indigenous groups (inc. Palauan, Samoan, Fiji & Soloman Islanders, Guatemalan)	4	6.0
*Year of Publication*		
< 1996	3	4.5
1996–2000	8	12.1
2001–2005	6	9.1
2006–2010	13	19.7
2011–2015	20	30.3
2016–2020	16	24.3
*Health Focus of Intervention*		
Diabetes prevention	19	28.8
Obesity prevention and treatment	12	18.2
Nutritional adequacy/Food security	9	13.6
Women and infant health	8	12.1
Diabetes management	7	10.6
Cardiovascular disease prevention	6	9.1
Chronic disease prevention (otherwise not already covered)	5	7.6
*Type of primary Nutrition Intervention component*		
Nutrition education	34	51.6
Healthy food environment	14	21.2
Individual dietary intervention	8	12.1
Cooking classes	4	6.0
Other (eg. Cultural stories, home-visits, dental care)	6	9.1
*Population*		
Adult	29	43.9
Child	18	27.3
Both	19	28.8
*Intervention duration*		
< 3 months	14	21.2
3–6 months	15	22.7
> 6–12 months	15	22.7
> 12–24 months	11	16.7
> 24 months	10	15.2
Not reported	1	1.5

**Fig. 2:**
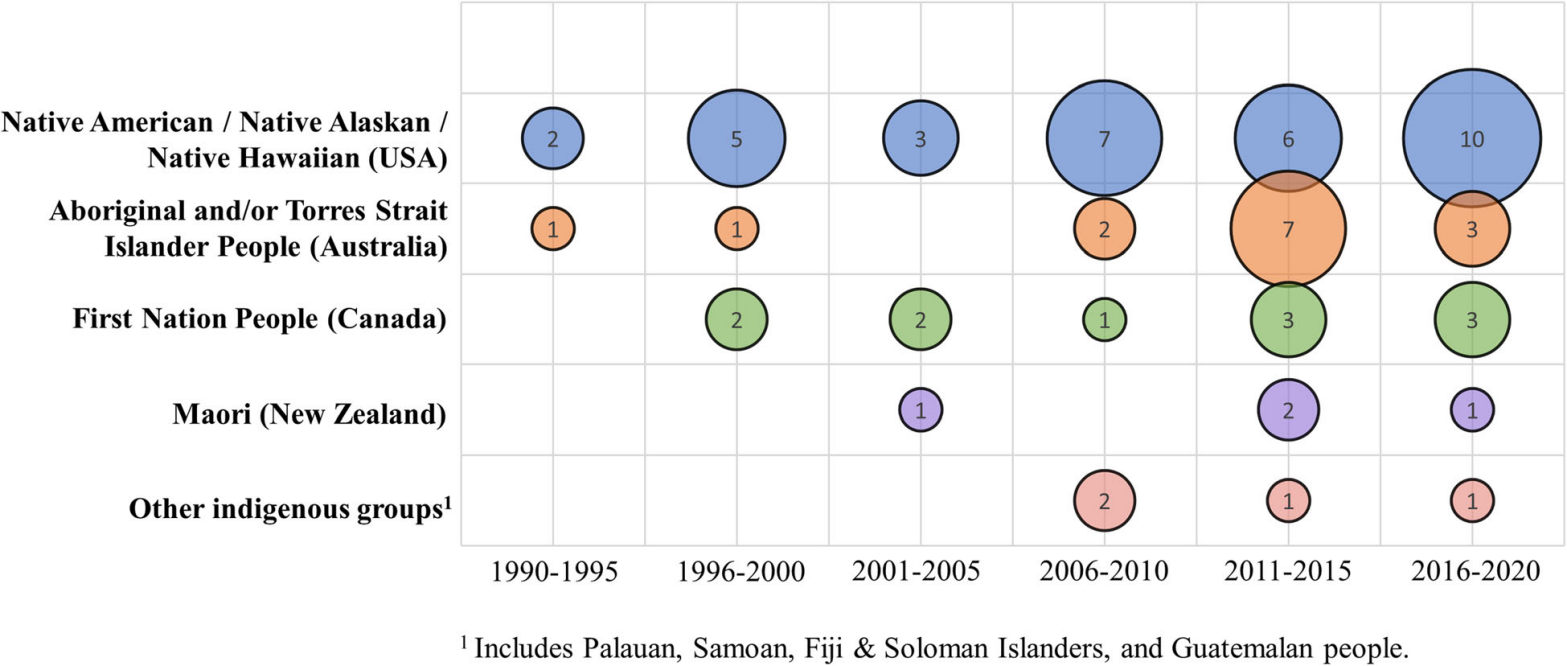
Bubble chart of interventions by publication year and by Indigenous population and location (*n* = 66)

The majority of interventions were conducted in the USA among Native American (***n*** **= 27**) [28, 32, 33, 37, 38, 42, 43, 46, 48, 50, 52, 56–58, 61–63, 65, 70, 72–74, 82, 83, 87, 89, 92], Alaskan (***n*** **= 4**) [47, 64, 75, 93] & Hawaiian (***n*** **= 2**) [54, 71] populations. Together with Australian Aboriginal and/or Torres Strait Islander (***n*** **= 14**) [27, 29–31, 40, 44, 51, 68, 78, 80, 85, 86, 88, 91] and Canadian First Nations People (***n*** **= 11**) [35, 36, 41, 45, 49, 55, 67, 69, 76, 84, 90] these three groups accounted for 85% of the literature included in this review. Figure 2 illustrates the interventions published by year and by Indigenous population and location.

**Fig. 3:**
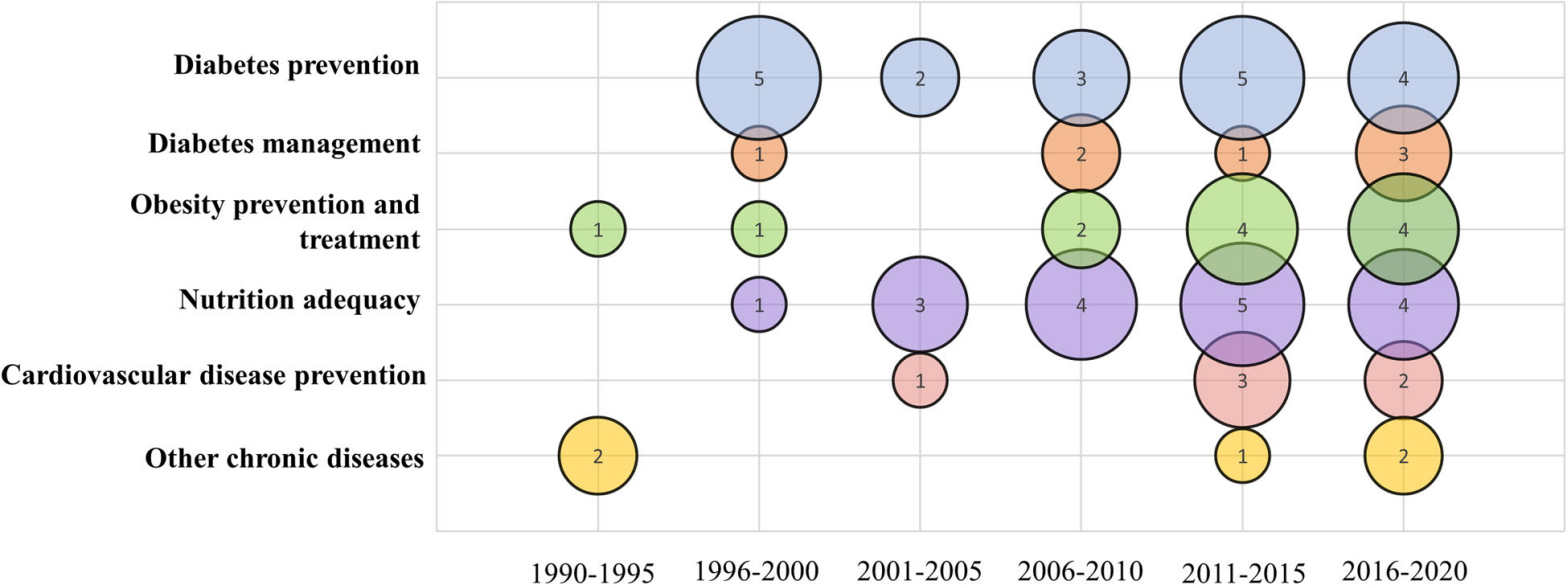
Bubble chart of articles published by Indigenous population and location and helath focus (*n* = 66)

Most interventions were designed for adults (***n*** **= 29**) [27, 28, 36, 39, 40, 42, 46, 51–54, 58, 60–63, 66, 71, 74–77, 80, 81, 83, 85–87, 91], with less than a third (***n*** **= 19**) [29–31, 34, 35, 38, 44, 47, 48, 50, 59, 65, 68, 69, 73, 78, 79, 88, 90] designed for both adults and children. Most interventions aimed to change health outcomes for diabetes prevention (***n*** **= 19**) [27, 28, 36, 38, 44, 55, 56, 60, 62, 67, 69, 70, 79, 81, 82, 88, 89, 92, 93], obesity prevention or treatment (***n*** **= 11**) [32, 34, 42, 50, 57, 59, 65, 72, 80, 83, 90], and diabetes management (***n*** **= 7**) [31, 33, 39, 48, 51, 58, 63, 68, 71, 86, 87, 91]. Figure 3 illustrates interventions published by Indigenous population and health focus.

### Intervention characteristics and cultural adaptation used in included studies

Table 3 summarises the intervention characteristics and cultural adaptation strategies employed (*n* = 66). Interventions that incorporated both individual and community level approaches were the most common (***n*** **= 32**) [28, 29, 33, 34, 37, 41, 44, 47, 49, 50, 52–56, 58–63, 66–68, 70, 71, 74–76, 88, 92, 93]. Two studies each employed community only [43, 90], system only [35, 57] and individual plus system [39, 91] level interventions. Seven interventions aimed to influence all three levels [30–32, 45, 69, 81, 89]. Most interventions **(*n*** **= 56)** [28, 30–39, 41–50, 52–59, 61, 63–69, 77, 80, 81, 83–87, 90–92] cited formative research. Only one-third of interventions (*n* = 22) were informed by a combination of formative research at both the population and community level [31–33, 35, 37–39, 42, 44, 46, 49, 54, 64, 66, 69–74, 86, 90]. Of the 46 studies that referred to formative research and also reported outcomes, most reported positive intervention outcomes (***n*** **= 38; 83%)** [28, 30–33, 36–39, 42, 43, 45, 47, 50, 53, 54, 56–58, 61, 63, 64, 66–73, 80, 84–86, 89–91, 93]. Nineteen interventions reported employing an underlying theory of behaviour change [28, 31–33, 36, 41, 42, 50, 55, 57, 65, 67, 70, 72, 73, 80, 90, 92, 93]. Of those that did, most employed multiple theories of behaviour change **(*n*** **= 9)** [31, 33, 36, 50, 55, 65, 90, 92, 93] social cognitive theory (***n*** **= 7**) [28, 41, 42, 57, 72, 73, 80], or its predecessor of social learning theory (***n*** **= 3**) [32, 67, 70].

**Table 3:** Summary of Intervention chracteristics and cultural adaptation strategies (*n* = 66)

Study Characteristic	n (%)	(%)
*Level of Intervention*		
Individual only	12	18.2
Community only	2	3.0
System only	2	3.0
Individual + Community	32	48.5
Individual + System	2	3.0
Community + System	9	13.6
Individual + Community + System	7	10.6
*Level of Community Input on design*		
None	16	24.2
Minimal	21	31.8
Maximal	29	43.9
Formative research cited		
Population level	28	42.4
Community level	6	9.1
Both	22	33.3
None	10	15.2
*Intervention based on Behavior change theory*		
None reported	47	71.2
Social cognitive theory	7	10.6
Social learning theory	3	4.5
Multiple therories	9	13.6
Number of cultural strategies employed^a^		
1 strategy	1	1.5
2 strategies	8	12.1
3 strategies	23	34.8
4 strategies	22	33.3
5 strategies	12	18.2
*Cultural sensitivities employed by category*^b^		
Surface only	12	19.7
Deep only	3	3.0
Both surface and deep	51	77.3

^a^Kreuter et al., 2003 [19] and ^b^Resincow et al., 1999 [20]

Almost all interventions employed more than one type of strategy to culturally tailor an intervention (***n*** **= 65**). **Twelve** interventions employed all five cultural adaptation strategies [34, 38, 48, 51, 53, 63–65, 68–70, 76], **22** employed four strategies [36, 39, 42, 44, 46, 47, 49, 54–61, 67, 72–74, 81], **23** employed three strategies [28–30, 32, 33, 37, 40, 41, 43, 45, 50, 52, 62, 71, 78, 82, 83, 86–89, 93], **eight** employed two strategies [27, 31, 35, 79, 80, 85, 91, 92], and only **one** study employed a single strategy [90]. Most interventions employed a combination of surface and deep cultural sensitivity approaches (***n*** **= 51)** [27–77], **12** used surface strategies only [78–89] and **three** studies [90–92] employed deep strategies only.

Cultural adaptation strategies used in each individual study are described in Additional file 3. Visual adaptation strategies were the most frequently used (***n*** **= 57**) such as ensuring print materials had pictures of Indigenous peoples, native foods, or colours of cultural significance [28, 29, 32–34, 36–49, 51–76, 78, 79, 81–89]. The second most frequently used strategy was constituent involving (***n*** **= 51**) which ranged from the surface level strategies of requesting participant feedback, incorporation of participant stories or engagement of local media and businesses (***n*** **= 20**) [27, 33, 36, 39, 42, 44, 46, 47, 61, 63, 68, 78, 80–84, 86–88], through to the deep level strategies of training of respected community members to deliver an intervention (***n*** **= 31**) [28–30, 32, 34, 37, 38, 41, 45, 48–51, 53, 55–60, 64, 65, 67, 69, 70, 72, 73, 90–92]. Socio-cultural strategies were also widely employed (***n*** **= 50**) and included a range of activities such as incorporating traditional activities and ceremonies in the intervention, and ensuring childcare was available for participants [27–47, 49–66, 68–71, 73–76, 91, 92]. Linguistic strategies were employed in 41 of the interventions. Most of these were at the surface level [34, 40, 42–44, 48–50, 52, 54, 57, 59, 62, 63, 68, 70, 75, 79, 81–84, 86–89], such as incorporating single Indigenous words into intervention materials (e.g. name of the intervention), or by translating messages exactly from English into Indigenous languages. Deep linguistic strategies (*n* = 15) included providing all materials in Native languages and English, or by incorporating Native language and concepts into the intervention materials and activities [38, 39, 51, 53, 60, 64–67, 69, 71, 72, 74, 76, 93]. Evidential strategies were least commonly employed (***n*** **= 34**) and included providing specific information about disease risk for Indigenous peoples in the community [30, 34–36, 38, 46–48, 51, 53–56, 58, 61, 63–65, 67–70, 72–76, 78, 80, 81, 84, 85, 89].

## Discussion

This is the first scoping review, to our knowledge, to examine the extent and range of research undertaken to culturally adapt nutrition interventions for Indigenous peoples across the world. Despite there being no restriction on year of publication, the review found only 98 papers representing 66 studies that fit the inclusion criteria. The publication rate on this topic appears to be accelerating with more than half of the included studies (*n* = 36) published in the past decade. Most studies were conducted with first peoples in the USA, Australia and Canada, despite the fact that Indigenous peoples inhabit over 90 countries worldwide [94]. This may be partly due to the shared experience of European colonization in these countries, and subsequently the need to sensitively adapt health care to improve cultural appropriateness [25, 95, 96]. Interventions included in this review had a strong emphasis on the prevention and management of diabetes and obesity. This is consistent with the known prevalence of metabolic disease which occurs at higher rates in Indigenous compared with non-indigenous populations [97–99], and important given these conditions are responsible for much of the gap in life expectancy and burden of disease between Indigenous and non-indigenous peoples [100]. It is important to note that the representation of research in low- and middle-income countries was clearly absent, as was a focus on the double burden of malnutrition experienced in many Indigenous peoples from these nations [101, 102]. Further research in this area is urgently needed.

The review shows that there has been a concerted effort to culturally adapt the design and delivery of nutrition interventions for Indigenous populations particularly in more contemporary research. The primary intervention strategy in over half of the studies was nutrition education, compared to less than one quarter of interventions aiming to improve the food environment. This tends to be consistent with nutrition interventions around the world despite the fact that nutrition knowledge, or lack of it, is usually not the underlying cause of health problems. This is particularly important to consider in Indigenous populations where social, economic and environmental inequalities pose significant challenges to health, rather than a lack of nutrition education [103, 104]. The United Nations Sustainable Development Goals [105] encompass aims relevant to improving Indigenous health including ending poverty and reducing inequalities. Interventions aiming to improve nutrition-related health in Indigenous populations need to incorporate strategies beyond nutrition education to address the social determinants of health at a community level [105, 106]. Less than a third of the included studies reported basing their intervention on a stated behaviour change theory. This phase of intervention design needs to be considered given the findings of a systematic review of the use of behaviour change theories in nutrition interventions by Rigby and colleagues [107]. That review showed interventions based on behaviour change theories were more effective at achieving health outcomes [107]. The Rigby review also found social cognitive theory/social learning theory to be the most commonly applied theories in designing nutrition interventions, consistent with the findings in our review. This reinforces the dominance of nutrition education as a key component of interventions. The use of models that focus more on environmental change maybe more appropriate in designing interventions for indigenous populations.

While the majority of interventions were applied across multiple levels, this review found that only half of the interventions incorporated a community level approach. This is despite the definition of health for Indigenous peoples consistently adopting a holistic view incorporating social concepts that are expressed and applied in the community [94]. In a review of three environmental intervention case studies addressing chronic disease prevention interventions in American Indians, Gittelsohn and Rowan concluded that strategies were more successful when multiple intervention levels including environmental approaches with a particular emphasis placed on partnering with local stakeholders to positively influence healthy behaviours were implemented [108].

Importantly, this review identified that the majority of interventions employed multiple cultural adaptation strategies as classified according to Kreuter and colleagues’ categories [19]. Most commonly this involved adopting peripheral and evidential strategies at a level that Resincow would classify as surface, which is consistent with interventions predominantly taking a nutrition education approach. Of interest was that deep linguistic approaches were least commonly utilised. A deep linguistic approach would involve making texts culturally relevant rather than simply a direct translation. Health education resources are often non-specific and lack cultural sensitivities for specific populations [109]. Further, direct language translations may lack cultural nuances putting cultural safety at risk. Health education resources need to be community-owned and incorporate cultural sensitivities to improve utiliastion and acceptance.

The majority of interventions involved key stakeholders at a surface level, and less than half involved constituents on a deep level. The review highlights that cultural adaptation of interventions to date has commonly involved engaging with Indigenous community stakeholders to elicit key understandings and experiences, for example, in conducting formative research. However, it was less common for interventions to be owned, delivered and/or directed by the community themselves. Similar findings to this review have also been reported in a recent scoping review evaluating community engagement in the design and implementation of chronic disease-based interventions for Indigenous populations [110]. In that review, Wali and colleagues reported that despite the agreed need to engage with Indigenous communities to support intervention engagement, few meaningfully consulted the community through all levels of intervention design and delivery [110]. Several authors have similarly argued that adopting participatory design approaches involving Indigenous communities as co-designers and decision-makers from the outset of intervention development is critical to achieve meaningful and lasting change [111–113].

A strength of this review was the application of an organisational system for categorising cultural adaption by a Kreuter and colleagues [19] which allowed for consistent and clear description of approaches currently used in nutrition interventions for Indigenous populations around the world. The additional application of Resnicow and colleagues [20] sensitivity levels to these categories has provided another analysis and categorisation of how interventions to date have been culturally adapted. This has highlighted areas for improvement in nutrition and health intervention research, particularly related to the need for deeper approaches that go beyond consultation, such as co-design. However, we note that while the classification systems by Kreuter and colleagues [19] and Resincow and colleagues [20] are published, they are by no means recognised as a combined validated tool to measure cultural adaptability. Constructing and validating a tool to guide cultural adaptation would be useful for the design of culturally safe interventions for Indigenous populations. Further, the classification schemes used are open to subjectivity in their interpretation and may therefore be influenced by the researcher during data extraction.

The review had other limitations. Given the significant differences between the cultures of Indigenous populations worldwide and the variety of health outcomes included, an evaluation of intervention effectiveness was not within the scope of this review. Determining intervention effectiveness by Indigenous population and health outcome is an important next step. Future evaluations of intervention effectiveness should consider the level of cultural adaptations made so as to better understand potential mechanisms for intervention success. Despite the rigourous search and screening methods, given the numerous terms used to describe Indigenous populations worldwide it is possible the search strategy was not able to capture all relevant studies. Further, Indigenous health research is often published in grey literature and subsequently may not have been identified for inclusion. While this review comprehensively investigated cultural adaptation strategies used, it did not evaluate the cultural safety of these interventions. Future research should consider the cultural safety of health interventions.

## Conclusion

This review is an initial step in overcoming the many barriers to developing and implementing culturally safe interventions. The results of this review provide evidence of what has previously been done and highlights priority areas for further research. This scoping review found that there is growing literature reporting complex and diverse approaches to cultural adaptation of nutrition interventions for Indigenous populations across the world. Interventions commonly use a mix of approaches for cultural adaptation, however few are adopting approaches that involve constituents at deeper levels where interventions can be owned, delivered and/or directed by the community themselves. The review highlights the need to move beyond traditional nutrition education techniques focused on behaviour change, to strengthen cultural adaptation approaches that involve Indigenous people at the community level as co-designers and decision makers in all phases of the intervention. Further research is now needed to explore the effectiveness of the types and levels of cultural adaptations used on various health outcomes to determine the most effective strategies for culturally safe nutrition interventions. This is especially relevant given the need to improve health equity for Indigenous populations and the increasing number of interventions being conducted in this population group.

## Supplementary Information

**Additional file 1.** Detailed summary of intervention characteristics

**Additional file 2.** Search strategy terms with example MeSH subject headings used in Medline and Scopus databases

**Additional file 3.** Detailed summary of cultural adaptation strategies, formative research and theories of behaviour change used

## Data Availability

All available data is included in the publication.

## Abbreviations

CINAHL: Cumulative Index to Nursing and Allied Health Literature
RCTs: Randomized Controlled Trials
USA: United States of America
