# Supplementary material for: Cultural adaptation of health interventions including a nutrition component in Indigenous peoples: a systematic scoping review
Source: Int J Equity Health. 2021 May 22;20:125. doi: 10.1186/s12939-021-01462-x (PMC8140502; doi:10.1186/s12939-021-01462-x)
Supplement: Supplementary file 1 — Additional file 1. Detailed summary of intervention characteristics [file 12939_2021_1462_MOESM1_ESM.docx]

**Additional File 1:** Detailed summary of intervention characteristics

| **Study;**  **First Author (yr)** | **Indigenous group (Region; Country)** | **Study design** | **Target group** | **Level of intervention**  **(tick)** | | | **Intervention** | | **Outcomes Reported?** | | | | | **If Yes, What Outcomes Changed?** | | |  |
| --- | --- | --- | --- | --- | --- | --- | --- | --- | --- | --- | --- | --- | --- | --- | --- | --- | --- |
|  |  |  |  | Individual | Family/ community | System | Description | Length (frequency and duration) | No | | | Yes | |  |  |  |  |
| ***Obesity Prevention*** | | | | | | | | | | | | | | | | |  |
| **Pathways**  Caballero (1998, 2003), Gittelsohn (1999) Davis (1999, 2003) | Native American  (AZ, MD, MN, NM; USA) | RCT | Children enrolled in a Native American school attending in yrs 3 – 5 (aged approx. 8-11 yrs) | X | X | X | I: School curriculum with increased physical activity at school, school food service improvements and, family involvement at school events.  C: Nil intervention | 3 yrs  (12 x 45 minute classroom lessons per yr, PA at 90 minutes per week) |  | | | X | | Increase in fruit and vegetable intake | | |  |
| **Navajo Healthy Stores**  Gittelsohn (2013) | Navajo  Native American  (AZ, NM, UT, USA) | Cluster RCT | Adults (≥18yrs) living on reservation, not pregnant, identified as the main food shopper for the household |  | X | X | I: Food stores conducted in store cooking demonstrations, taste testing of healthy foods, promotional give-aways, and installed healthy food promotions, shelf nutrition labels and educational displays. Local media disseminated healthy messages.  C: waitlist intervention for 6-10 weeks | 1 yr |  | | | X | | Reduction of BMI.  Increased purchase of health food options. | | |  |
| **Project Y.E.A.H**  Hemmingson (2016) | Native American (SD, USA) | Pre-post (pilot) | College students (≥18 years old) attending South Dakota State University | X |  |  | I: Interactive website with written education focused on behaviour change, healthful eating, physical activity, and stress management  C: Pre-intervention data | Ongoing | X | | |  | |  | | |  |
| **NAP SACC (The Nutrition and Physical Activity Self-Assessment for Child Care)**  Mattingly (2016) | Oglala Lakota  Native American (ND, USA) | Pre-post | Children (aged 2-5 yrs) attending child care  And;  Child centre staff on Pine Ridge Indian Reservation |  |  | X | I: Training workshop provided for child care centre staff including cooks on child nutrition and how to encourage healthy eating behaviours. Child care centre staff chose specific action goals to carry out at their workplace including access to water, access to healthy foods, and healthy food education. Brochures on healthy eating and physical activity for children were provided to parents and caregivers  C: pre intervention data | 1 day workshop, and 1 yr action plan |  | | | X | | Policies implemented.  Attitudes of child care workers improved. | | |  |
| **OPREVENT** (Redmond, 2019) | Native American (USA) | Cluster RCT | 5 Tribal communities (adults and families) across the Upper Midwest & Southwest US |  | X | X | I: Education provided & physical changes made to school, food store and worksite environments. Media also used to share info. Intervention conducted across 5 phases & focused on nutrition and physical activity.  C: Delayed intervention | Five phases completed over 20 weeks total (each component had differing lengths) | X | | |  | |  | | |  |
| **Bright Start**  **(ohiyu lyojanjan)**  Story (2012) | Lakota  Native American (ND & SD, USA) | Cluster RCT | Children (aged 5 – 7 years) attending school in the Lakota schools district |  | X | X | I: Based on Pathways intervention. Physical activity at least 60 mins per day at school, School provided lunches with more fruits and vegetables and reduced access to sugar sweetened beverages. Family events held at the school included meals, physical activities and behavioural change messages for home. Parents received 3 x motivational encouragement phone calls from trained research staff to set behavioural goals for the family.  C:waitlist intervention (time not specified) | 24 months (frequency and duration not reported) |  | | | X | | Fewer % in overweight category. | | |  |
| ***Obesity Treatment*** | | | | | | | | | | | | | | | | |  |
| **SHARE-AP ACTION** Anand  (2007) | Six Nations Reservation,  First Nations Peoples (Canada) | RCT | Families of at least one parent and one child over 5 years of age living on Six Nations Reservation, willing to have home visits. |  | X |  | I: AHW’s deliver tailored diet and PA goal setting to household, encouraged and organised grocery store tours, label reading, cooking classes, and sports  C: Basic written guidelines for healthy diet and PA (usual care). | 6 months (frequency not reported) |  | | | X | | Decrease in total energy and weight in both groups. No difference between groups. | | |  |
| **Aboriginal and Torres Strait Islander Women’s Fitness Program**  (Canuto 2011 & Canuto 2012) | Indigenous Australian (Urban Adelaide, SA, AUS) | RCT | Women (18-64 yrs) with no pre-existing medical conditions (including pregnancy), living in Adelaide metropolitan area. | X |  |  | I: Structured exercise class, physical activity diary with prompts for 10,000 steps per day, Dietitian led group education sessions which covered basic nutrition, label reading, recipe modification, and healthy takeaways.  C: Waitlist intervention for 12 months. | 12 weeks  (Weekly 45-60 minute exercise, 4 x 60 minute Dietitian led education sessions) |  | | | X | | Decrease in weight and BMI and sustained at 3 months after intervention. | | |  |
| **Fit Kit Palau**  Collier (2018) | Palauan (Palau, Micronesia) | Pre-post | Teenagers and adults (15+ yrs), overweight or obese | X | X |  | I: Written education materials that could be transformed into oral lessons about nutrition, physical activity, sleep and mindfulness.  C: None. | 8 weeks  (weekly sessions, duration not reported) | X | | |  | |  | | |  |
| **Healthy Children, Strong Families**  LaRowe (2007), Adams  (2012) & Tomayko (2016)  **Healthy Children, Strong Families 2 (HCSF2)**  Tomayko (2017 & 2018) | Oneida Tribe, Menominee, Lac du Flambeau and Bad river,  Native American  (WI, USA) | RCT | Families with at least one parent, and one child between 2 and 5 yrs, registered as members of participating tribes. | X | X |  | I^1^: Individual, family, and group education on fruit and vegetable intake, sugary foods and beverages, physical activity and screen time. Tailored goal setting for parents and families during home visits with trained mentors (respected community members), group support meetings.  C^1^: Mailed written lifestyle information in the same topics as intervention.  I^2^: 12 monthly mailed lifestyle intervention lessons and children’s books (Wellness Journey). Emphasis on increasing F&V intake & activity, decreasing screen time, sugar and improving sleep quality. Educational materials accompanied by supportive text messages & online group forum.  C^2^: Intervention focused on child safety (Safety Journey) | 24 months (12 months tailored family support, 12 months group support; frequency not reported) |  | | | X | | I^1^: Negligible changes in weight, dietary intake or physical activity.  I^2^: Improved overall diet quality, increased F&V intake and physical activity | | |  |
| ***Project REPLACE***  *Mercer (2013)* | Maori, (NZ) | Pre-post case studies | Targeted at overweight or obese individuals, but available for any person. | X | X |  | I: Community based programs tailored to the needs of the specific community. All programs were in the theme of REPLACE  **R**egular exercise  **E**at health food  **P**articipate  **L**ose weight  **A**lcohol reduction  **C**ut out smoking  **E**ducate  Some examples of community programs were access to a 24 hr gym only for obese participants in the program, or a community garden available to all members of the community.  C: pre intervention data | 12 weeks | X | | |  | |  | | |  |
| ***Diabetes Prevention*** | | | | | | | | | | | | | | | | |  |
| **Unnamed** Abbott  (2012) | Aboriginal Australians (Sydney, AUS) | Open RCT | Adults (aged ≥17 yrs) within the Aboriginal Medical Service of Western Sydney district. | X |  |  | I: Open TAFE course, run by local indigenous woman, and supported by AHW’s, focused on cooking skills and food buying on a budget for  C: none | 18weeks  4hrs/wk (72hours) | X | |  | |  | | |  |  |
| **Unnamed**  Allen (2008)  & Thompson (2008) | Native American (New Mexico, USA) | RCT | Asymptomatic women (aged 18-40 yrs), with impaired fasting blood glucose, not pregnant, living in metropolitan area of New Mexico. | X | X |  | I: Group education led by respected community leaders focused on food label reading, how to choose healthier foods, recipes, taste testing of healthy meals, 15 minutes physical activity, individual goal setting and follow up at the next session.  C: Waitlist intervention for 18 months. | 6 months (2-2.5 hours per month) |  | | X | | Decreased fasting blood glucose, decreased LDL, decreased total energy intake.  No change in weight. | | |  |  |
| **Unnamed**  Brooking (2012) | Maori (unspecified territory, NZ) | RCT | Community members <75 yrs, at risk of diabetes, but without any diagnosed chronic disease and waist circumference indicating high risk. | X |  |  | I: High protein, or high fibre dietary prescription. Provided nutrition information individually and in groups including written information, cooking classes, and shopping tours.  C: usual diet, waitlist for dietary support after 24 weeks | 24 weeks (weekly for 16 weeks, and as requested for the final 8 weeks) |  | | X | | Decrease in weight.  No change in fasting blood glucose or insulin levels. | | |  |  |
| **Journey to Native Youth Health**  (Brown, 2013) | Northern Plains reservation communities  Native American  (MT, USA) | RCT | Youths (10-14 yrs) at risk of developing diabetes | X | X |  | I: Education delivered by respected tribal community members, based on the DPP curriculum, with cultural components added such as traditional activities and foods (e.g. berry picking, dancing, hunting).  C: Education delivered by respected tribal community members focussed on alcohol and drug prevention. | 3 months  (9 sessions, time not reported), |  | | X | | No change in BMI or physical activity.  Improved knowledge, attitudes and behaviour about nutrition score. | | |  |  |
| **Ngati and Healthy**  Coppell (2009) & TIpene-Leach (2013) | Ngati Porou  Maori  (East Coast, NZ) | Pre-post | Adults (25+ yrs) attending Ngati Porou Hauora (health service) | X | X | X | I: Community health promotion programs, community nutrition education classes, and individual and family education for those identified at risk. | 2 yrs |  | | X | | No changes in fasting blood glucose, insulin resistance, dietary intake or physical activity. | | |  |  |
| **The Okanagan Diabetes Project**  Daniel (1999) | Okanagan, Penticton and Splats’in  First Nations Peoples (British Colombia, CAN) | NRT | Adult (18 yrs old +) community members |  | X | X | I: Community health promotion activities for physical activity and nutrition, Local media distributed educational material on healthy foods, exercise and weight loss and shared success stories of community members.  C: community without interventions, but continued usual care. | 24 months |  | | X | | Increased HbA1c, decreased BMI and blood pressure.  No changes in diet or exercise behaviours. | | |  |  |
| **The Traditional Foods Program (TFP)**  DeBruyn (2020) | Native Americans  (USA) | Pre-post | All community members across 17 tribal groups in the US |  | X | X | I: Community engagement through activities covering general topics focused on traditional foods (ie. Gardening, hunting), physical activity and social support. Each tribal group designed their own intervention/s based on their group’s cultural values  C: Baseline | 5 years, with bi-annual data collection (total of 10 collection points) |  | | X | | No health outcome data reported.  Increased # of community gardens, access to traditional foods, physical activity from baseline to T10 | | |  |  |
| **AI Youth Wellness Camp**  Gachupin (2017 & 2019) | Native Americans (AZ, USA) | Pre-post | Youths (10-15 yrs old) attending a wellness camp | X |  |  | I: Daily education sessions on healthy eating, physical activity and disease prevention.  24hr dietary recalls, anthropometric and behaviour assessments  C: Baseline data | 1 week |  | | X | | Total fat intake decreased. | | |  |  |
| **Diabetes Management and Care Program**  Gracey (2006) | Aboriginal  Australian  (WA, AUS) | Pre-post | All community members in remote Northern WA. | X | X |  | I: Education sessions for local schools on healthy diet and physical activity for children with input from indigenous sporting celebrities. Community health screening program through local health clinics to screen for obesity, dyslipidaemia and diabetes.  Community health promotion programs for physical activity and diet.  C: pre-intervention data | Ongoing | X | |  | |  | | |  |  |
| **Special Diabetes Program for Indians Diabetes Prevention (SDPI -DP)**  Jiang, 2013 & 2018  **Enhanced SDPI (Special Diabetes Program for Indians)**  Rosas (2016 & 2020) | Native Americans, Alaskan Natives  (USA) | Pre-post  RCT | Adults (≥18 yrs old) with pre-diabetes or diabetes | X | X |  | I1: Group education on healthy lifestyle behaviours, plus individual lifestyle coaching sessions to set goals and solve barriers.  C1: pre intervention data, historical data.  I2: Standard SDPI with 3 additional enhancements: i) talking circles for social support; ii) photovoice activity for diabetes self-management; iii) digital story sessions  C2: Standard SDPI (special diabetes program for Indians) | 10 yrs  (24 x weekly classes, duration not reported + quarterly individual session for 10 yrs, duration not reported)  12 months (16 x weekly group education sessions; outcome data collection at 0, 6 and 12 months) |  | | X | | Lower incidence of diabetes.  Decreased weight, blood pressure, fasting blood glucose levels, blood lipids.  No difference between groups for primary or secondary outcome measures.  Control group had lower consumption of unhealthy foods compared to intervention group | | |  |  |
| **Kahnawake Schools DPP**  Macaulay (1997), Paradis (2005) | Mohawk  First Nations Peoples (QC, CAN) | NRT | Elementary school children (ages 5-11 yrs) attending school in the Kahnawake schools district. | X | X |  | I: Health Education curriculum introduced for grades 1 through 6 at all schools in the Kahnawake district, based on the diabetes prevention program. Nutrition covered healthy foods, the roles of nutrients in the body, label reading, healthy habits, factors influencing healthy eating habits, and foods commonly eaten in the community. Physical activity included benefits of daily activity and the different types of activity. A section on diabetes and its consequences and prevention was included. Story telling, games, try it out activities and crafts were incorporated to all lessons.  C: Usual school health curriculum | 3 yrs |  | | X | | No changes in BMI, dietary intake or physical activity. | | |  |  |
| **STOP diabetes! (Students Teaching Others to Prevent Diabetes)**  Marlow (1998) | Winnebago and Omaha  Native American  (NE, USA) | Pre-post | Adolescents (aged 13-18 yrs) living on the Winnebago Reservation. | X | X |  | I: Four adolescents designed and delivered a half day program that included cultural stories, and legends as a framework for discussing healthful aspects inherent in traditional Native American life, food preparation and tasting, and time for anthropometric measurements with personalised advice for physical activity. Written information on diabetes, exercise and nutrition was provided for all participants.  C: pre intervention data | 4 hours workshop;  8 x 3-4 hours for program development. |  | | X | | Increase in knowledge.  Change in diabetes risk not reported. | | |  |  |
| **Unnamed**  Murphy (2003); McAuley (2003) | Maori (NZ) | Pre-post | Adults (aged 24-60 yrs) without diagnosed diabetes. | X | X |  | I: Individualised dietary and exercise plan for each participant with a focus on traditional foods and traditional exercise. Participants were invited to group exercise 4 times per week and a healthy cooking class once per month for 3 months.  C: pre intervention data | 12 weeks |  | | X | | Increased insulin sensitivity.  Decreased weight, waist circumference, BMI, and blood pressure. | | |  |  |
| **Pima Action & Pima Pride Interventions**  Narayan (1998) | Pima or Akimel Oʼodham  Native American (AZ, USA) | NRT | Overweight, normoglycemic adults (aged 25-53 yrs) in the Gila River Indian Community. | X | X |  | I1: Calorie restriction and physical activity supported by individual sessions with dietitian and group meetings. Meetings included food preparation demonstrations, grocery store tours and group problem solving.  I2: Self-directed learning facilitated by an appreciation of Pima Culture. Small group meetings held once per month to discuss lifestyles, culture and history. Participants received printed information on healthy diet, and exercise habits. | 12 months |  | | X | | Increased physical activity in both groups.  Decreased dietary intake of energy, fats, and carbohydrates in I2.  No changes in weight, fasting blood glucose, or blood pressure. | | |  |  |
| **Healthy Buddies – First Nations version**  Ronsley (2013) | Tsimshian Nation  First Nations Peoples (BC, CAN) | Cluster NRT | Children attending school (K-12, aged approx. 5-18 yrs), on Tsimshian Nation Reservation | X | X |  | I: School curriculum updated to include education on Physical activity, healthy eating and healthy body image. Fitness sessions include vigorous circuit type activities. Older students are buddied with younger students to teach and model healthy behaviours.  C: No curriculum changes or buddy system. | 10 months (21 education and 6 fitness sessions, 30 minutes each) |  | | X | | Decreased zBMI and waist circumference.  Increased blood pressure in control group.  No change in dietary intake or physical activity. | | |  |  |
| **Sandy Lake Health and Diabetes Project**  Saksvig (2003) & Kakekagumick (2013) | Ojibway, Cree  First Nations People  (ON, CAN) | Pre-post | All community members in Sandy Lake Reserve. | X | X | X | I: Incorporated multiple substantiated programs such as Northern Store Program (Vastin & Curran), a home visit program (refs x 4), local radio diabetes information segments, and tailored school based curriculums that included nutrition education, physical activity and diabetes specific education (MacAulay), as well as community diabetes prevention programs (Jiang, Brown)  C: pre-intervention data | 3 yrs and ongoing (frequency and duration determined at class level) |  | | X | | Increased BMI, waist circumference, % body fat, self efficacy and nutrition knowledge scores  Decreased screen time, and VO_2_ max. | | |  |  |
| **Tribal turning point**  Sauder (2017) | Cherokee and Navajo Nations  Native American (OK, USA) | RT | Identified tribal children (aged 7 – 10 yrs), overweight or obese, without diabetes, with at least 1 parent to participate. | X | X |  | I: Group education on physical activity, cultural activities cooking and nutrition, Motivational interviewing with child and caregiver, and informational ‘toolbox’ including recipes, community events calendar, and health service contacts.  C: Equal timed education and written ‘toolbox’ on general health and wellbeing, including bullying, first aid, drug and alcohol prevention. | 8 months  (10 sessions, 2 hours each with 10-20 mins physical activity. 5 motivational interviews, duration not reported) |  | | X | | Decrease in BMI z-score.  No changes in blood pressure, fasting glucose level, fasting insulin levels or HbA1c. | | |  |  |
| **Maboo Wirriya, Be Healthy**  Seear (2019) | Small rural community (Derby)  (WA, Australia) | Pre-post | Young (15-25 yrs old) Aboriginal community members at high risk of diabetes | X | X |  | I: Group sessions including cooking classes, exercise circuits and education aligned with diabetes prevention program.  C: Pre-intervention data | 2 months (8 weekly sessions, 1.5 hours long with 30 mins education and practical skills) | X | |  | | N/A | | |  |  |
| **Zuni Diabetes Prevention Program**  Tuefel (1998) | Zuni  Native American (NM, USA) | Pre-post | High school aged children (aged approx.. 13-18 yrs) attending school in the Zuni school district | X | X | X | I: Established a teen wellness facility, School curriculum updated with diabetes education (including increased physical activity and nutrition), removal of sugary beverages from schools, addition of fruit and fruit products (frozen fruit bars) at school.  C: pre-intervention data | 2 yrs and ongoing |  | | X | | Decrease in BMI, decrease in sugar sweetened beverage consumption. | | |  |  |
| ***Diabetes Treatment*** | | | | | | | | | | | | | | | |  |  |
| **Flinders Model of Self-management support**  Battersby (2008) | Wangka Wilurrara  Indigenous Australians  (SA, AUS) | Pre-post | Aboriginal patients with diabetes, aged ≥40yr in the Ceduna or Port Lincoln area | X |  | X | I: AHWs developed and delivered a new model of care which focussed on improved self-management planning, goal setting, and care coordination by AHW’s rather than GPs  C: pre-intervention data | 12 months (frequency not reported) |  | | X | | Decrease in HbA1c and Diabetes assessment score.  No change in blood pressure. | | |  |  |
| **Together on diabetes program**  Chambers (2015), Kenney (2016) | Navajo and White Mountain Apache tribe.  Native American  (AZ, USA) | Pre-post | Youth (10-19 yrs old) identified with T2DM, pre-diabetes, or identified as ‘at risk of T2DM’ | X | X |  | I: family home based visits by a health coach to educate the individual separate health coach education for a support person, regular clinic appointments, and community events for diabetes prevention.  C: None. | 12 months  (12 x 1 hr sessions for 6 months, 6 x 20 minute sessions for 6 months + 4 x 30 minute sessions for a nominated support person in 12 months) |  | | X | | Decreased BMI and blood pressure.  No change for HbA1c, or dietary intake. | | |  |  |
| **Diabetes Care in American Samoa**  DePue (2013 & 2013) | Samoan  (American Samoa, Polynesia) | Cluster RT | Adults (≥20 yrs) with T2DM and no treated comorbidities (e.g. kidney failure) attending the local health service | X |  | X (new prioritisation algorithm) | I: New treatment algorithms identified required patient frequency and intensity based on risk. Patients received guided goal setting by a cultural health worker and written material adapted from the National Diabetes Education Program. Group education was available for identified ‘high risk’ patients. | 1 yr  (frequency and duration determined individually) |  | | X | | Decreased HbA1c.  No change in blood pressure, weight, or waist circumference. | | |  |  |
| **Medicine Wheel Nutrition Intervention** Kattleman (2009) | Cheyenne River Souix  Native American (SD, USA) | RCT | Community members (age not reported) with T2DM, and no comorbidities (e.g. kidney failure) or planned treatments (e.g. alcohol treatment). | X |  |  | I: Group nutrition education based on the medicine wheel nutrition model, delivered by a dietitian and respected tribal member. Individualised meal plans based on medicine wheel model that met energy and macronutrient requirements.  C: Waitlist intervention for post trial (timing not reported). | 6 months (monthly 2 hour sessions, individual advice frequency and duration not specified) |  | | X | | No change in BMI, HbA1c, blood lipids, blood pressure, dietary intake or physical activity. | | |  |  |
| **FEDS (The Family Education DIabettes Series)**  Mendenhall (2010) | Native American (MN, USA) | Pre-post | Adults (22-80 yrs) living in urban areas of Minneapolis or St Paul with diabetes and without current diabetes treatment. | X | X |  | I: Group education with patients, family members, health care providers and village elders. Sessions include meal preparation, portion sizes, diabetes and healthy weight maintenance, stress management, diabetes complications and prevention. Specific session content is informed by the group each week.  C: pre intervention data | 6 months (fortnightly, 3-4 hours each) |  | | X | | Decreased blood pressure, decreased weight.  No change in HbA1c. | | |  |  |
| **Keya Tracker** Robertson (2007) | Native American (Northern Plains, AL, USA) | NRT | Native Alaskan adults (aged ≥18 yrs) with T2DM and without renal failure. | X |  |  | I: Website with information on healthful lifestyles and cultural traditions. Information on physical activity, nutrition, cultural and medical activities (e.g. diabetes group therapy, wellness gatherings etc) was collected. Immediate feedback provided on physical activity, calories and grams of protein, fat, saturated fat, carbohydrates, fibre, protein, cholesterol and sodium.  C: Usual care | 24 weeks (3 sessions per week, duration not specified) |  | | X | | No change in dietary intake, weight, exercise or HbA1c. | | |  |  |
| **Looma Healthy Lifestyle**  Rowley (2000 & 2001) | Looma Community,  Aboriginal Australians (WA, AUS) | NRT | Aboriginal people (15+ yrs), overweight or obese, or with diagnosed diabetes.  After 2 yrs, the weight and diabetes criteria was dropped. | X | X |  | I: Group physical activities such as hunting, sport, and walking groups. Shopping tours, cooking demonstrations and formal diabetes education sessions. Optional body weight and blood glucose checks.  C: No intervention. | 24 months (weekly sessions, duration not reported) |  | | X | | Increase in fruit and vegetable intake, and plasma antioxidants.  Decrease in Total-Chol among 15-35 yr olds only.  No change in fat intake. | | |  |  |
| ***Chronic Disease (unspecified) Prevention*** | | | | | | | | | | | | | | | |  |  |
| **Stores Healthy Options at Remote Indigenous Communities (SHOP-RIC)**  Brimblecomb (2013, 2017 & 2018) | Indigenous Australians (NT, AUS) | Stepped RCT | All members of rural and remote Northern Territory indigenous community members across 20 communities | X | X | X | I: 20% discount on fruits and vegetables was instigated at community food stores associated with Arnhem Land Progress Aboriginal Corporation and Outback Stores. Price drops were followed in half the intervention groups by in store nutrition education posters.  C: Waitlist intervention by up to 8 months. | 6 months (frequency N/A) |  | | X | | Increased purchase of fruits, vegetables, water, and other “healthy options”.  Decreased purchase of sugar sweetened beverages. | | |  |  |
| **Survival Tucker**  Lee (1994) | Aboriginal Australians (NT, AUS) | NRT | Adults (≥18 yrs) in the Minjilang community (Croker Island, 240km North east of Darwin) |  | X | X | I: Provision and promotion of nutritious foods in local shops, introduction of ‘shelf-talkers’ to aid recognition of good foods, broad community wide encouragement to exercise.  C: No intervention | 12 months |  | | X | | Decreased BMI, purchase of high fat foods, high sugar foods.  Increased blood concentration of antioxidants and carotenoids, and purchase of fruits and vegetables. | | |  |  |
| **Home-based Kidney Care**  Nelson (2018) | Zuni Indians  (New Mexico, USA) | RCT | Zuni Indians (21-80 yrs old) with chronic kidney disease | X | X |  | I: Bi-weekly home visits to provide education sessions on: diet, exercise, alcohol, smoking and chronic disease management.  C: Usual care | 12 months (data collection at 6 month intervals) |  | | X | | Increased patient activation  Decreased BMI, HbA1c, CRP | | |  |  |
| **Aboriginal Get Healthy Service**  Quinn (2017) | Aboriginal Australians  (NSW, AUS) | Pre-post | Aboriginal Ausn adults (aged ≥18 yrs) without chronic disease, and not requiring physician input | X |  |  | I1: Phone support line with 13 sessions of individually tailored health coaching with goal setting for physical activity and healthy diet  I2: Three written information packs to promote healthy lifestyles including increased physical activity and healthy dietary patterns. | 6 months (frequency and duration determined by participant) |  | | X | | Decreased weight, BMI, waist circumference, intake of sugar sweetened beverages.  Increased exercise and intake of fruits and vegetables. | | |  |  |
| **The Waianae Diet program** (Shintani, 1991 & 1994) | Native Hawaiian (HI, USA) | Pre-post | Native Hawaiian adults (aged ≥18 yrs), overweight or obese, with or without chronic disease (CVD, diabetes, cancer) | X | X |  | I: Non-calorie restricted diet high in locally grown, traditional Hawaiian foods such as taro, fern shoots, fruit, seaweed, fish and chicken. Daily education sessions, with traditional meal provided were held for 21 days.  C: pre-intervention data | 21 days (frequency and duration of education not reported) |  | | X | | Decreased HbA1c, serum glucose and blood lipids, weight, and caloric intake. | | |  |  |
| ***Nutritional Adequacy*** | | | | | | | | | | | | | | | |  |  |
| **Pathways to prevention project**  Foley (2011) | Aboriginal and Torres Strait Islander  (QLD, AUS) | Pre-post | Adults (≥18 yrs) | X |  |  | I: Cooking classes based on “Deadly Tucker” recipes. Specific recipes were selected by each group. After cooking, the meal was shared by the group and discussions were held on where and how to buy food economically.  C: None | 3-4 weeks  (weekly 3-4 hour sessions) | X | |  | | Not reported | | |  |  |
| **Harvest Sharing Program**  Gates (2016) | First Nations Peoples  (Ontario, CAN) | Pre-post | Youth (12 – 15 yrs) in rural Ontario | X | X |  | I: Adults with appropriate licenses were identified by the local community council, and supported financially to harvest lesser snow geese, and share them with the community.  C: Historical data | 3 months |  | | X | | No changes in dietary intake. | | |  |  |
| **Unnamed**  Govula (2007) | Lakota  Native American (ND&SD, USA) | Pilot RCT | Children attending school on or near reservation, enrolled in yr 3 (8 yrs old). |  | X |  | I: Nutrition curriculum added to classroom,. Topics were based on MyPyramid and the Medicine Wheel Nutrition for Native Americans, delivered by a dietitian.  C: Waitlist intervention for 6 weeks | 6 weeks  (1 x 30 minute lesson per week) |  | | X | | Increased nutrition knowledge and intake of vegetables. | | |  |  |
| **Earthbox Kids Garden Education**  Hanbazaza (2015) | Kipohtakaw  First Nations Peoples (AB, CAN) | Pre-post | Children attending school in Alexander First Nation reserve, enrolled in yrs 1 – 6 (5-10 yrs old). | X | X | X | I: Food gardening kits incorporated into the curriculum by the teachers. Excess produce from the gardens were used for the school snack program. The school snack program provided a variety of fruits and vegetables prepared in different ways for children to taste.  C: pre-intervention data | 2 yrs (frequency and duration determined by teacher); data collected at baseline, 7mo and 18mo |  | | X | | Increased intake, preference and knowledge about fruits and vegetables | | |  |  |
| **THRIVE study**  Jernigan (2018); | Chicksaw, Choctaw Nations  Native American (OH, USA) | Cluster RCT | Adults (≥18 yrs old) who are tribal citizens of Chicksaw or Choctaw nations. |  | X | X | I: Increased availability of fruits, vegetables, nuts/seeds, wholegrains, and healthy snacks, enhanced placement of healthy food choices, promotion of healthy foods, and reduced price of healthy food.  C: Waitlist for intervention (unspecified time) | 9 months (Nation A) and  12 months (Nation B) |  | | X | | No change in fruit or vegetable intake within group or between groups | | |  |  |
| **Traditional Food Program**  Kenny (2018) | Inuit, Athapaskan  Indigenous peoples  (Inuvik, CAN) | Pre-post | Children (13-18 yrs) enrolled in local schools | X | X |  | I: Yr 7-12 curriculum incorporated identification, growing, harvesting, preparation and cooking of traditional foods. Community gatherings allowed for traditional foods to be consumed, as well as further learning on the origin and importance of traditional foods from Elders.  C: pre-intervention data | 1 yr and ongoing  (weekly) | X | |  | |  | | |  |  |
| **Uli’eo Koa Program (Warrior Preparedness program)**  Leslie (2001) | Native Hawaiian Peoples (Hawaii, USA) | Pre-post | Native Hawaiian adults (22-60 yrs of age) who are moderately active and not obese. | X | X |  | I: 3 weeks of group structured exercise and pre-prepared meals (provided) based on the traditional Hawaiian diet; followed by 8 weeks of less frequent structured exercise and only 2 pre-prepared meals provided pet week. | 11 weeks  (60 minutes, twice/day for three weeks, 60 minutes three/week for eight weeks). |  | | X | | Increased intake of protein, fibre, vitamins A, C and E, calcium and iron.  Decreased intake of saturated fat | | |  |  |
| **Fish-to-School (Neqa Elicarvigmun)**  Nu (2017);  Bersamin (2019) | Yup’ik  Native Alaskan (AL, USA) | Pre-post | indigenous youths attending school (12-18 yrs of age) in Native Alaskan communities |  | X | X | I: Provision of traditional salmon once per week in school lunches for children in middle & high school, curriculum to include cultural pride and food systems, and community wide events such as fish scavenger hunts, and food themed film festival.  C: pre-intervention data | 9 months (baseline, 4 month & 9 month data collection) |  | | X | | Improved diet quality;  Increased fish intake among intervention group | | |  |  |
| **Apache Healthy Stores**  Vastine (2005), Curran (2005) | San Carlos and White Mountain Apache tribes  Native American (AZ, USA) | Pre-post | All community members living on 2 reservations. |  | X | X | I: Increased availability of healthy foods in stores, healthy food promotions in store and on local media, in store cooking demonstrations.  C: historical data | 1 yr |  | | X | | Increased food knowledge, healthy food purchases and intention to eat healthy foods. | | |  |  |
| ***Maternal and Infants Health*** | | | | | | | | | | | | | | | |  |  |
| **Nutrition Awareness Project – Failure to Thrive**  Balmer (1997) | Ngaanyatjarra Pitjantjatjara Yankunytjatjara  Aboriginal Australians  (Central (SA, WA, NT, AUS) | Pre-post | Young mothers and children | X | X |  | I: Education for mothers, crisis intervention at hospitals, written information packages for new mothers and education for community food stores.  C: pre-intervention data | Not reported | X | |  | |  | | |  |  |
| **Aboriginal Maternity Group Practice Program**  Bertilone (2015) | Indigenous Australians  (South Metropolitan Health District, Perth, WA, AUS) | NRT | All Aboriginal women who gave birth while participating in the AMGPP from July 2011 to December 2012 | X | X | X | I: Team based care that included midwives, AHW’s, and well respected grandmothers from the community, all provided support, antenatal education, attended health appointments, and helped to navigate health system.  C1: usual antenatal care during same time frame (2011-2012) as I group  C2: historical data from mothers who gave birth between 2009-2011. | 18 months (frequency determined at patient level) |  | | X | | Decreased number of preterm babies, likelihood of neonatal resuscitation at birth, and overall LOS | | |  |  |
| **Smiles not Tears**  Blinkhorne (2014), Smith (2018) | Indigenous Australians (NSW, AUS) | Pre-post | Families from 8 rural, remote or metropolitan areas of NSW with at least one child >6months old | X |  |  | I: Leaflets and fridge magnets developed by health care experts outlined good oral health behaviours for children and families. Materials were provided to mothers of young children (<5 yrs) through AHW’s, schools, the local ACCHO, and other local indigenous centres.  C: historical data | 30 months  (frequency N/A) |  | | X | | Fewer caries in the intervention group | | |  |  |
| **TOTS (toddler overweight and tooth decay prevention study)**  Karanja (2010) | Native American, Alaskan Native,  (ID, OR, WA, USA) | Pre-post | Expectant mothers and their families from 3 tribes | X | X |  | I: Home visits by health workers to promote and assist breastfeeding, and replace sugar sweetened beverages with water.  C:local media, brochures and health promotion activities to reduce sugar sweetened beverage consumption. | 12 months  (7-21 home visits, duration not reported) |  | | X | | Increased breastfeeding rate and duration over time.  Decreased rate of weight gain. | | |  |  |
| **Your baby’s smile**  Lawrence (2004) | Ojibway, Cree,  First Nations People (ON, Can, Arctic Circle,) | Pre-post | Children aged 2-5 yrs from 16 communities around the Sioux Lookout Zone | X |  |  | I: Written education pamphlets, educational messages reinforced by local media promotion and food store tours for new mothers and prenatal women. Pamphlets and referrals for the Head Start Brushing program for toddlers by community dentists and reinforced during Well Child Checks.  C: historical data | 2 yrs |  | | X | | Increased parental knowledge.  No change in number of childhood caries. | | |  |  |
| **Si Yo Estoy Bien, Mi Familia También (If I am OK, my family is too)**  Leenen et al (2008) | Guatemalan Indigenous peoples (GT) | NRT | Women from rural Guatemala who were part of local health programs | X | X |  | I: Four group education modules on health, nutrition and food for women, hygiene and sanitation, as well as sexuality and reproductive health. Cultural elements, and agency for women were woven throughout the program modules. Women who completed the program were offered a position as a health promoter to distribute the program further in the community.  C: no intervention | 15 x 3 hr (180hours) PLUS 4 days paid training for health promoters. |  | | X | | Increased knowledge and agency.  Increased variety and completeness of diet. | | |  |  |
| **Baby Teeth Talk**  Merrick (2012), Smithers (2017)  Jamieson (2018) | Ausn Indigenous peoples (SA, AUS) | RCT | Pregnant mothers expecting an Indigenous baby or a new mother to an Indigenous baby <6 weeks old | X |  |  | I: from 6 months old, includes dental care (cleaning, extractions, restorations), Fluoride varnish, Motivational interviewing and education of mother (including nutrition education on non-carious foods), and anticipatory guidance for mother for the following 18months.  C: waitlist for treatment until child is 24 months. | 2 yrs |  | | X | | Decreased number of caries, and decreased number of damaged teeth.    No change in dietary intake of children. | | |  |  |
| **Close to the Heart**  Murphy (2008) | Native American (AZ, US) | Pre-post | Native women who were in the process of making choices about breastfeeding initiation | X | X |  | I: Tribal members of different ages and genders shared personal stories, and traditional stories that highlight the value of breastfeeding in a video. Participants are also provided specially created calendars, posters and brochures to promote breastfeeding. Daily breastfeeding rounds by lactation consultation or breastfeeding technician to support new mothers initiate BF.  C: pre intervention data | 12 minute video, and once off materials |  | | X | | Increased proportion of women breastfeeding beyond 8 weeks. | | |  |  |
| ***Cardiovascular Disease*** | | | | | | | | | | | | | | | | |  |
| **Unnamed**  Counil (2012) | Nunavik Inuit  First Nations’ people  (Nunavut, CAN) | Pre-post | All persons in Nunavik communities |  |  | X | I: Public health nutritionists worked with Nunavik leaders to introduce food labelling of transfats, and to reduce number of items containing transfats at food stores and restaurants  C: historical data | 3 yrs and ongoing | X |  | | | | | Not reported | | |
| **Unnamed**  Davis (1993) | Navajo, Pueblo  Native American  (NM, USA). | Pre-post | Children attending schools in Pueblo and Navajo regions enrolled in fifth grade (aged approx. 11 yrs) | X | X |  | I: Education focussed on physical activity, basic nutrition, tobacco use, habit change, and social influences. Written information sent home to parents on cardiovascular health. Homework included bringing the lessons to the community (e.g. video for the senior centre)  C: waitlist intervention for | 12 months (18 hours total class time, plus homework) |  | X | | | | | Increased knowledge, and physical activity.  Decreased use of butter.  No change in BMI | | |
| **The Balance Study**  Lee (2012); | Apache, Caddo, Comanche, Delaware, Ft. Stil Apache, Kiowa and Wichita  Native American (OK, USA) | RCT | Adults (aged 30-75 yrs), with metabolic syndrome, legally identified as part of Apache, Caddo, Comanche, Delaware, Ft. Stioll Apache, Kiowa, or Wichita tribes | X | X |  | I: Group education and individual sessions for goal setting. Each meeting included a weigh in, review of cultural, spiritual, emotional, nutritional and physical wellbeing, and provision of a healthy meal. Dietary advice was based on the 2006 diet and lifestyle recommendations for CVD risk reduction.  C: written information on healthy living from the American Heart Association, in addition to written materials provided to the intervention group | 24 months (fortnightly meetings for one yr, monthly meetings for one yr. Duration not reported) | X |  | | | | |  | | |
| **Unnamed**  Rolleston (2016 & 2017) | Maori (NZ) | Pre-post | Maori adults (aged ≥18 yrs) with no history of CVD events, but at least 2 CVD risk factors | X | X |  | I: Group exercise classes, education sessions [including nutrition education and cooking demonstrations], and informal meetings between group members and family members. All participants received individual physical activity prescriptions and tailored dietary advice.  C:pre intervention data | 12 weeks  (exercise 3/week, education 1/fortnight) |  | X | | | | | Decreased waist circumference, hip circumference, blood pressure, and HDL-C.  Increased QoL.  No change in weight or total Chol | | |
| **Healthy Hearts Across Generations**  Walters (2012) | Native American  (Northwest, USA) | RT | Adult tribal members (≥18 yrs), overweight or obese with any other health conditions under control, with at least one dependent child around 5 yrs of age. | X | X |  | I1:Family goal setting, commitment, and revision followed by motivational interviewing and tailored family support (e.g. running coach, shopping tour). Group physical activity and nutrition skills building workshops.  I2: Family goal setting, commitment and revision followed by motivational interviewing and tailored family activities such as scrap books or photo journals, kite making, drum making and basket weaving. | 12 months  (weekly goals and motivational interviewing, duration not reported; weekly workshops, 2-3 hours) | X |  | | | | | Not reported | | |
| **Traditions of the heart**  Stefanich (2005), Witmer (2004), Hiratsuka (2007) | Native American (Alaska, USA) | RCT | Adult Native Alaskan women (aged 40+ yrs) | X | X |  | I: Small group education for physical activity, stress management, tabaco, and nutrition including cooking demonstrations ,food models, traditional foods, and available foods. Individual goal setting sessions. Cookbook with heart healthy meals and traditional foods.  C: Delayed intervention (time not specified) | 12 weeks (2 hours per week) |  | X | | | | | No change in BMI, blood lipids, blood pressure, or intake of fruits and vegetables.  Increase in physical activity. | | |
| **Seven Sisters**  Ziabakhsh (2016) | First Nations Peoples (BC, CAN) | Pre-post | Indigenous women working as health advocates or recognised as Elders. | X | X |  | I:Group social and educational sessions. All sessions included talking circles and imparting wisdom from elders, traditional ceremonies, and nutritious food. Education included physical activity, smoking, nutrition, culture in physical health, and mental health.  C: pre-intervention data | 8 weeks  (2 hours per week) | X |  | | | | | Not reported | | |

C: Control Group; I: Intervention Group; RCT: randomised controlled trial; RT: Randomised trial: NRT: nonrandomised trial; CVD: Cardiovascular Disease;BP: Blood pressure, HDL-C: High density Lipoprotein-Cholesterol; LDL-C: Low density Lipoprotein-Cholesterol; Total-Chol: Total Cholesterol; HbA1c: glycolated haemoglobin; approx.: approximately; yrs: years; year: yr.
