# Supplementary material for: Cultural adaptation of health interventions including a nutrition component in Indigenous peoples: a systematic scoping review
Source: Int J Equity Health. 2021 May 22;20:125. doi: 10.1186/s12939-021-01462-x (PMC8140502; doi:10.1186/s12939-021-01462-x)
Supplement: Supplementary file 2 — Additional file 2. Search strategy terms with example MeSH subject headings used in Medline and Scopus databases [file 12939_2021_1462_MOESM2_ESM.docx]

**Additional File 2:** Search strategy terms with example MeSH subject headings used in Medline and Scopus databases

| Populations | Aborigin* OR “Torres Strait” OR Indigen* OR “Native American” OR “Alaska Native” OR “Hawaiian Native” OR “Native Hawaiian” OR “Native Alaskan” OR “First Nation” OR “First People” OR Maori OR Inuit OR “American Indian” OR “Pacific Island” OR [sh] “Oceanic Ancestry Group” [sh] OR American Native Continental Ancestry Group OR [sh] OR Health Services [sh] OR Indigenous [sh] |
| --- | --- |
| Food and Nutrition | Nutrition* OR Food* OR Diet* OR Eat* OR Cook* |
| Treatment | Intervention* OR Therap* OR treat* OR Program* OR Manage* |

[sh]= subject heading
