# Supplementary material for: Cultural adaptation of health interventions including a nutrition component in Indigenous peoples: a systematic scoping review
Source: Int J Equity Health. 2021 May 22;20:125. doi: 10.1186/s12939-021-01462-x (PMC8140502; doi:10.1186/s12939-021-01462-x)
Supplement: Supplementary file 3 — Additional file 3. Detailed summary of cultural adaptation strategies, formative research and theories of behaviour change used [file 12939_2021_1462_MOESM3_ESM.docx]

**Additional File 3:** Detailed summary of cultural adaptation strategies, formative research and theories of behaviour change used

| **Study Name**  First Author (year) | **Formative research cited?** Yes/No | | **Theory of behaviour change employed** | **Community members involved in design of intervention** | **Type of strategies employed to culturally tailor an intervention** | | | | | | |
| --- | --- | --- | --- | --- | --- | --- | --- | --- | --- | --- | --- |
|  |  |  |  |  | Evidential | Peripheral | Linguistic | | constituent-involving | | Socio-cultural  (deep level only) |
|  | Population level | Community level | Yes/No | Yes/No | Surface | Surface | Surface | Deep | Surface | Deep | Deep |
| ***Obesity Prevention*** | | | | | | | | | | | |
| **Pathways**  Caballero (1998, 2003), Davis (1999, 2003) Gittelsohn (1999) | Yes | Yes | Yes - social learning theory | Yes - maximal |  | X |  |  |  | X | X |
| **Navajo Healthy Stores**  Gittelsohn (2013) | Yes | Yes | Yes - Social Cognitive theory, social marketing | Yes- maximal |  | X | X |  | X |  | X |
| **Project Y.E.A.H**  Hemmingson (2016) | Yes | No | Not reported | Yes - minimal |  | X | X |  | X |  |  |
| **NAP SACC (The Nutrition and Physical Activity Self Assessment for Child Care)**  Mattingly (2016) | Yes | No | Yes - Social Cognitive Theory | No |  | X | X |  |  | X | X |
| **OPREVENT** (Redmond, 2019) | No | Yes | Yes – Social Cognitive Theory & Social Ecological Model | Yes - maximal | X | X |  | X |  | X | X |
| **BrightStart (ohiyulyojanjan)**  Story (2012) | Yes | Yes | Yes - Social cognitive theory | No | X | X |  | X |  | X |  |
| ***Obesity Treatment*** | | | | | | | | | | | |
| **SHARE-AP ACTION**  Anand (2007) | Yes | Yes | Yes - Protection motivation theory, social learning theory normative influences and theories of persuasion | No |  |  |  |  |  | X |  |
| **Aboriginal and Torres Strait Islander Women’s Fitness Program**  Canuto (2011 & 2012) | Yes | No | Yes – social cognitive theory | Yes - minimal | X |  |  |  | X |  |  |
| **Fit Kit Palau**  Collier (2018) | No | Yes | Not reported | Yes - maximal | X | X | X |  |  | X | X |
| **Healthy Children, Strong Families (HCSF)**  LaRowe (2007), Adams (2012), Tomayko (2016)  **Healthy Children, Strong Families 2 (HCSF2)**  (Tomayko 2017 & 2018) | No | Yes | Yes - Social cognitive and family systems theory | Yes - maximal |  |  | X |  |  | X | X |
| **Project REPLACE**  Mercer (2013) | Yes | No | Not reported | Yes – minimal |  | X | X |  |  | X | X |
| *Diabetes Prevention* | | | | | | | | | | | |
| **Unnamed**  Abbott (2012) | No | No | Not reported | No |  |  |  |  | X |  | X |
| **Unnamed**  Allen (2008) & Thompson (2008) | Yes | No | Yes - social cognitive theory | Yes - minimal |  | X |  |  |  | X | X |
| **Unnamed**  Brooking (2011) | No | No | Not reported | No |  | X | X |  |  |  |  |
| **Journey to Native Youth Health**  Brown (2013) | Yes | No | Yes - social cognitive theory, trans-theoretical stages of behaviour change | Yes - maximal |  |  |  |  |  | X | X |
| **Ngati and Healthy**  Coppell (2009)  Tipne-Leach (2013) | Yes | No | Not reported | No | X | X | X |  | X |  |  |
| **The Okanagan Diabetes Project**  Daniel (1999) | No | Yes | Yes - social learning theory, theory of reasoned action, health belief model, community change model | Yes - maximal | X | X |  |  | X |  | X |
| **The Traditional Foods Program (TFP)**  DeBruyn (2020) | Yes | Yes | Not reported | Yes - maximal | X | X |  | X |  | X | X |
| **AI Youth Wellness Camp**  Gachupin (2017 & 2019) | No | No | Not reported | Yes - minimal |  | X | X |  | X |  |  |
| **Diabetes Management and Care program**  Gracey (2006) | Yes | Yes | Not reported | Yes - maximal |  | X | X |  | X |  | X |
| **Special Diabetes Program for Indians Diabetes Prevention (SDPI-DP)**  Jiang (2013 & 2018)  **Enhanced SDPI-DP**  Rosas (2016 & 2020) | Yes | No | Yes - social cognitive theory, trans-theoretical stages of behaviour change | Yes - minimal | X |  |  | X |  |  | X |
| **Kahnawake Schools DPP**  Macauley (1997)  Paradis (2005) | Yes | No | Yes - Social Learning Theory, Ottowa Charter for Health Promotion, traditional learning styles of native children | Yes – maximal | X | X |  |  |  | X | X |
| **STOP Diabetes**  Marlow (1998) | Yes | No | Not reported | Yes – maximal | X | X |  |  |  | X | X |
| **Unnamed**  Murphy (2003); Macauley (2003) | No | No | Not reported | Yes - maximal |  | X |  | X |  | X | X |
| **Pima Action & Pima Pride Interventions**  Narayan (1998) | No | No | Not reported | No |  | X | X |  |  |  | X |
| **Healthy Buddies – First Nations version**  Ronsley (2013) | Yes | No | Yes - Social learning theory | Yes – minimal | X | X |  | X |  | X |  |
| **Sandy Lake Helath and Diabetes Project**  Saksvig (2003) & Kakekagumick (2013) | Yes | Yes | Not reported | Yes - minimal | X | X |  | X |  | X | X |
| **Tribal turning point**  Sauder (2017) | Yes | Yes | Yes - Social learning theory | Yes – maximal | X | X | X |  |  | X | X |
| **Maboo Wirriya, Be Healthy**  Seear (2019) | No | No | Not reported | Yes - minimal |  | X | X |  | X |  |  |
| **Zuni Diabetes Program**  Tuefel (1998) | Yes | No | Not reported | No | X | X | X |  |  |  |  |
| *Diabetes Treatment* | | |  |  |  |  |  |  |  |  |  |
| **Flinders Model of Self-management support**  Battersby (2008) | No | Yes | Not reported | Yes - maximal |  |  |  |  |  | X | X |
| **Together on diabetes program**  Chambers (2015), Kenney (2016) | Yes | Yes | Yes – ecological and trans-theoretical stages of behaviour change. | Yes - maximal |  | X |  |  | X |  | X |
| **Diabetes Care in American Samoa**  DePue (2013 & 2013) | Yes | Yes | Not reported | No |  | X |  | X | X |  | X |
| **Medicine Wheel Nutrition Intervention**  Kattelmann (2009) | Yes | No | Not reported | No | X | X | X |  |  | X |  |
| **FEDS (The family education diabetes series)**  Mendenhall (2010) | Yes | No | Not reported | Yes – maximal | X | X |  |  |  | X | X |
| **Keya Tracker**  Robertson (2007) | Yes | No | Not reported | Yes - minimal |  | X | X |  | X |  |  |
| **Looma Healthy LIfestyle**  Rowley (2000 &2001) | Yes | No | Not reported | Yes – maximal | X | X | X |  | X |  | X |
| *Chronic Disease prevention* | | |  |  |  |  |  |  |  |  |  |
| **Stores Healthy Options at Remote Indigenous Communities (SHOP-RIC)**  Brimblecomb (2013, 2017 &2018) | Yes | Yes | Yes (Social ecological theory and social cognitive theory) | Yes – minimal |  | X |  |  |  |  | X |
| **Survival Tucker**  Lee (1994) | No | No | Not reported | Yes – maximal | X | X |  | X |  | X | X |
| **Home-based Kidney Care**  Nelson (2018) | No | Yes | Not reported | Yes - maximal | X | X | X |  | X |  | X |
| **Aboriginal Get Healthy Service**  Quinn (2017) | Yes | Yes | Not reported | Yes – minimal |  | X | X |  | X |  |  |
| **The Waianae Diet program**  Shintani (1991 & 1994) | Yes | Yes | Not reported | Yes - maximal |  | X |  | X |  |  | X |
| ***Nutritional Adequacy*** | | |  |  |  |  |  |  |  |  |  |
| **Pathways prevention project**  Foley (2011) | No | No | Not reported | No |  | X | X |  |  |  | X |
| **Harvest Sharing Program**  Gates (2011) | Yes | No | Yes - social cognitive theory | No |  | X |  |  |  | X | X |
| **Unnamed**  Govula (2007) | Yes | No | Not reported | Yes - minimal |  | X | X |  |  |  | X |
| **Earthbox Kids Garden Education**  Hanbazaza (2015) | Yes | No | Not reported | No |  | X |  |  |  | X | X |
| **THRIVE study**  Jernigan (2018); | Yes | Yes | Not reported | Yes - minimal | X | X |  |  | X |  | X |
| **Traditional food program**  Kenny (2018) | Yes | Yes | Not reported | Yes - maximal |  | X | X |  |  | X | X |
| **Uli’eo Koa Program (Warrior Preparedness progam)**  Leslie (2001) | Yes | Yes | Not reported | No | X | X | X |  |  |  | X |
| **Fish-to-school (Neqa Elicarvigmun)**  Nu (2017);  Bersamin (2019) | Yes | Yes | Not reported | Yes – maximal | X | X |  | X |  | X | X |
| **Apache Helathy Stores**  Vastine (2005), Curran (2005) | Yes | Yes | Yes - Social Cognitive theory | Yes - maximal | X | X |  |  |  | X | X |
| ***Maternal and infant health*** | | |  |  |  |  |  |  |  |  |  |
| **Nutrition Awareness Project – Failure to thrive**  Balmer (1997) | No | No | Not reported | Yes - maximal |  | X |  |  |  | X | X |
| **Aboriginal Maternity Group Practice program**  Bertilone (2015) | Yes | No | Not reported | Yes - maximal | X |  |  |  |  | X | X |
| **Smiles not Tears**  Blinkhorn (2014), Smith (2018) | No | No | Not reported | Yes - minimal | X | X |  |  | X |  |  |
| **TOTS (toddler overweight and tooth decay prevention study)**  Karanja (2010) | Yes | No | Not reported | No | X | X |  |  | X |  | X |
| **Your baby’s smile**  Lawrence (2004) | Yes | No | Not reported | Yes - minimal | X | X | X |  | X |  |  |
| **Si Yo Estoy Bien, Mi Familia Tambien (If I am OK, my family is ok)**  Leenen (2008) | Yes | No | Not reported | Yes – minimal | X | X |  | X |  | X | X |
| **Baby Teeth Talk**  Merrick (2012)  Smithers (2017)  Jamieson (2018) | Yes | No | Not reported | No | X | X |  |  |  |  |  |
| **Close to the Heart**  Murphy (2008) | Yes | No | Not reported | Yes – maximal | X | X |  |  | X |  | X |
| ***Cardiovascular Disease prevention*** | | | | | | | | | | | |
| **Unnamed**  Counil (2012) | Yes | Yes | Not reported | Yes - minimal | X |  |  |  |  |  | X |
| **Unnamed**  Davis (1993) | Yes | Yes | Not reported | Yes - minimal |  | X |  |  |  | X | X |
| **The Balance Study**  Lee (2012) | Yes | No | Not reported | Yes – maximal |  | X | X |  |  |  | X |
| **Unnamed**  Rollerston (2016 & 2017) | Yes | Yes | Not reported | Yes – maximal |  | X |  | X |  |  | X |
| **Healthy Hearts Across Generations**  Walters (2012) | Yes | No | Not reported | Yes- maximal | X | X |  | X |  |  | X |
| **Traditions of the heart**  Stefanich (2005), Witmer (2004), Hiratsuka (2007) | Yes | Yes | Not reported | Yes – minimal | X | X | X |  |  |  | X |
| **Seven sisters**  Ziabakhsh (2016) | Yes | No | Not reported | Yes – minimal | X | X |  | X |  | X | X |
